# Intravenous Fosfomycin: An Assessment of Its Potential for Use in the Treatment of Systemic Infections in Canada

**DOI:** 10.1155/2018/8912039

**Published:** 2018-06-25

**Authors:** George G. Zhanel, Michael A. Zhanel, James A. Karlowsky

**Affiliations:** Department of Medical Microbiology and Infectious Diseases, Max Rady College of Medicine, University of Manitoba, Winnipeg, MB, Canada

## Abstract

Fosfomycin is a bactericidal agent that inhibits cell wall synthesis using a mechanism of action distinct from *β*-lactams or other antimicrobial agents. It is a broad-spectrum agent that is frequently active against antimicrobial-resistant bacterial pathogens including methicillin-resistant *Staphylococcus aureus* (MRSA), vancomycin-resistant enterococci (VRE), multidrug-resistant (MDR) Enterobacteriaceae, and some isolates of MDR *Pseudomonas aeruginosa*. Intravenous fosfomycin has been prescribed for a wide variety of infections in many countries for >40 years. It is most frequently used in combination with other antimicrobial agents (e.g., *β*-lactams, carbapenems, and aminoglycosides) and has an excellent safety profile, including in neonates and children, even with long-term administration (weeks). Fosfomycin achieves extensive tissue distribution including difficult to reach compartments such as aqueous humor, vitreous humor, abscess fluid, and CSF. Available data, to date, suggest no clinically relevant pharmacological interactions between fosfomycin and other agents, including drugs, stimulants, or food. Intravenous fosfomycin's role in therapy in Canada is likely as an agent used alone or in combination for complicated urinary tract infections in hospitalized patients as well as hospitalized patients with MDR infections who have not responded to first-, and potentially, second-line antimicrobials or in patients who cannot tolerate (due to adverse effects) first- and second-line antimicrobials.

## 1. Current State of Antimicrobial-Resistant Infections in Canada

### 1.1. Context: Global Trends in Antimicrobial Resistance

Infections among hospitalized patients in Canada, and worldwide, are increasingly reported to be attributable to antimicrobial-resistant, multidrug-resistant (MDR), and extensively drug-resistant (XDR) bacterial pathogens [1–6]. These pathogens include, but are not limited to, methicillin-resistant *Staphylococcus aureus* (MRSA), both community-associated MRSA (CA-MRSA) and healthcare-associated MRSA (HA-MRSA), vancomycin-resistant enterococci (VRE), penicillin-resistant *Streptococcus pneumoniae*, extended-spectrum *β*-lactamase (ESBL)-producing *Escherichia coli* and *Klebsiella pneumoniae*, fluoroquinolone-resistant and carbapenem-resistant Enterobacteriaceae (CRE), MDR *Pseudomonas aeruginosa*, and MDR *Acinetobacter* spp. [1–6]. Treatment options for antimicrobial-resistant, MDR, and XDR bacterial pathogens can be limited. Therefore, ongoing development of new agents, ideally with novel, bactericidal mechanisms of action and reliable activity against antimicrobial-resistant bacterial pathogens, is essential. Strong antimicrobial stewardship programs are also vital to curbing the emergence and spread of antimicrobial-resistant pathogens and to the future of successful infectious diseases patient care.

### 1.2. The CANWARD Surveillance Study: An Annually Trended and Current Perspective on Antimicrobial Resistance in Canadian Hospitals

For more than a decade, the Canadian Antimicrobial Resistance Alliance-CARA/Health Canada partnered CANWARD surveillance study has reported on the prevalence of antimicrobial-resistant bacterial pathogens in Canadian tertiary care hospitals. CANWARD annually collects clinical isolates from a network of participating laboratories across Canada and performs standardized Clinical and Laboratory Standards Institute (CLSI) defined broth microdilution antimicrobial susceptibility testing on the isolates in a central (coordinating) clinical microbiology laboratory [6–9]. From 2007 to 2016, CANWARD determined the in vitro activities (e.g., MIC_50_, MIC_90_, and MIC range) of dozens of marketed antimicrobials against ∼43,000 clinical isolates from blood (43.5% of isolates), respiratory (33.1%), urine (13.2%), and wound (10.2%) specimens [6]. Patient demographic parameters associated with these isolates included the following: patient's gender: male (54.6% of isolates) and female (45.4%); patient age: ≤17 years (13.0%), 18–64 years (44.3%), and ≥65 years (42.7%); and patient location: medical and surgical wards (38.1%), emergency rooms (24.8%), clinics (18.1%), and intensive care units (19.0%).

The CANWARD surveillance study has determined that the most common bacterial pathogens isolated from patients attending Canadian tertiary care hospitals (due to community acquired or healthcare-acquired infections) over the last decade (2007–2016) were *S. aureus* (21.2%, includes both methicillin-susceptible *S. aureus* (MSSA) and methicillin-resistant *S. aureus* (MRSA)), *E. coli* (19.5%), *P. aeruginosa* (9.0%), *S. pneumoniae* (6.1%), *K. pneumoniae* (6.1%), *Enterococcus* spp. (5.4%), *Haemophilus influenzae* (4.1%), and coagulase-negative staphylococci (3.8%) (Table 1) [6].

Antimicrobial susceptibility rates among Gram-positive cocci identified in CANWARD from 2007 to 2016 were highest for linezolid, daptomycin, and vancomycin, whereas for Gram-negative bacilli, susceptibility rates were highest for colistin, tigecycline, amikacin, ceftolozane-tazobactam, and meropenem [6, 7]. Among the most common pathogens, MRSA susceptibility rates were 100% for linezolid, 99.9% for vancomycin and daptomycin, 99.2% for tigecycline, 97.5% for doxycycline, 94.1% for trimethoprim-sulfamethoxazole, and 53.9% for clindamycin. *Streptococcus pneumoniae* susceptibility rates were 100% for vancomycin, 99.4% for ceftriaxone, 99.1% for moxifloxacin, 86.7% for doxycycline, 82.6% for penicillin, and 78.4% for clarithromycin. The most active agents against *E. coli* were meropenem, imipenem, and tigecycline (all >99.9% susceptible); colistin (99.8% using the European Committee on Antimicrobial Susceptibility Testing (EUCAST) MIC breakpoint); ertapenem and amikacin (both 99.7%); ceftolozane-tazobactam and piperacillin-tazobactam (both 97.6%); cefepime (95.1%); for ceftazidime (93.7%); ceftriaxone (91.8%); gentamicin (90.5%); for ciprofloxacin (77.9%); and trimethoprim-sulfamethoxazole (73.2%) [6]. Similarly, *P. aeruginosa* antimicrobial susceptibility rates for the most active agents in vitro were 98.3% for ceftolozane-tazobactam, 95.0% for colistin, 92.0% for amikacin, 84.2% for piperacillin-tazobactam, 82.9% for ceftazidime, 81.2% for meropenem, 79.7% for gentamicin, and 75.4% for ciprofloxacin [6]. For *Acinetobacter baumannii*, antimicrobial susceptibility rates for the most active agents in vitro were 97.5 for colistin, 95.9% for amikacin, 94.7% for meropenem, and 92.4% for ciprofloxacin [6, 7].

### 1.3. MRSA in Canada

For MRSA, CANWARD has documented a significant decrease in prevalence (% MRSA among *S. aureus*) from 26.1% in 2007 to 19.3% in 2011, with 28.9% of MRSA being community-associated CA-MRSA genotypes and 68.6% healthcare-associated HA-MRSA genotypes [10]. The prevalence of CA-MRSA genotypes significantly increased from 19.7% to 36.4% between 2007 and 2011 (*P* < 0.0001), with CA-MRSA10 (USA300) being the predominant CA-MRSA genotype. The hVISA phenotype was detected in 7/27 (25.9%) isolates of MRSA with a vancomycin MIC of 2 *μ*g/mL [10].

### 1.4. VRE in Canada

For VRE, CANWARD reported a Canadian prevalence of 4.2% (80/1927), with a significant increase from 1.8% in 2007 to 6.0% in 2013 [11]. All 80 VRE isolates were identified as *E. faecium*, with 90% carrying the *vanA* gene and 10% the *vanB* gene. All VRE were resistant to ciprofloxacin, levofloxacin, and vancomycin; 70.6%, 86.3%, and 100% of VRE were susceptible to doxycycline, linezolid, and daptomycin, respectively [11]. Treatment options for infections caused by VRE are currently limited.

### 1.5. ESBL-Producing *E. coli* and *K. pneumoniae* in Canada

In CANWARD, the prevalence of ESBL-producing *E. coli* and *K. pneumoniae* increased significantly from 3.4% to 12.6% and from 1.5% to 6.7%, respectively, from 2007 to 2016 [8, 9]. Approximately 31% of ESBL-producing *E. coli* displayed an MDR phenotype with concomitant resistance to cephalosporins, fluoroquinolones, aminoglycosides, and trimethoprim-sulfamethoxazole [8]; 0.8% of ESBL-producing *E. coli* were XDR (defined as concomitant resistance to ≥5 different antimicrobial classes). The majority of ESBL-producing *E. coli* in Canada, and elsewhere, are clonal (ST131) and carry the ESBL gene, CTX-M-15; the spread of the ST131 clone is also responsible for the majority of the observed increase in fluoroquinolone resistance among ESBL-producing *E. coli* over the last decade [8]. The most active agents against ESBL-producing *E. coli* were colistin, meropenem, and tigecycline (all >99.9% susceptible) along with ertapenem, amikacin, and ceftolozane-tazobactam (all ∼98% susceptible) [6, 7, 9, 10]. CANWARD has also recently identified the appearance of and slow increases in carbapenem resistance among clinical isolates of *E. coli* and *K. pneumoniae* (e.g., KPC-3) [8, 9].

### 1.6. MDR *P. aeruginosa* in Canada

MDR isolates of *P. aeruginosa* (i.e., concomitant resistance to ≥3 different antimicrobial classes) currently account for 5–10% of isolates of *P. aeruginosa* in CANWARD and demonstrate reduced susceptibility to many antipseudomonal agents, including tobramycin (92.6% susceptible (for all isolates) versus 51.3% susceptible (for MDR isolates)), piperacillin-tazobactam (85.1% versus 18.4%), meropenem (83.5% versus 16.5%), ciprofloxacin (77.9% versus 13.4%), and ceftazidime (83.7% versus 11.4%); in contrast, colistin is equally active against all isolates of *P. aeruginosa* and against MDR subsets (97.5% versus 98.1%) [12]. Ceftolozane/tazobactam displayed 99.0% susceptibility against all isolates of *P. aeruginosa* and 89.2% against MDR isolates [12].

## 2. Current Need for New Antimicrobial Agents in Canada

### 2.1. Need for New Antimicrobial Agents for Infections Caused by Resistant Gram-Positive Cocci

The most urgent need for new antimicrobials for Gram-positive pathogens in Canada is to address infections such as respiratory, bacteremic (including endocarditis), complicated skin and skin structure infections, and bone and joint infections that are associated with *S. aureus*, and specifically, infections caused by MRSA. *S. aureus* (both MRSA and MSSA) rank first among the most common pathogens in Canadian hospitals (Table 1), and MRSA represents ∼20% of all *S. aureus* isolates. Systemic MRSA infections in the hospital setting are currently treated with vancomycin; however, poor treatment outcomes, concern about the development of hVISA, MIC creep, pharmacokinetic limitations regarding certain compartments such as the central nervous system, and high rates of nephrotoxicity with increasing doses of vancomycin (to maintain serum trough levels at 15–25 *μ*g/mL and/or AUC >400 *μ*g·hr/mL) have clinicians reassessing vancomycin's role. Linezolid is active in vitro but is a bacteriostatic agent and is accompanied by hematological toxicities with prolonged use and drug interactions, whereas daptomycin cannot be used for MRSA respiratory infections due to inactivation by lung surfactant.

There is also an urgent need for new antimicrobials in Canada to address urinary tract, bacteremic, and wound infections associated with *Enterococcus* spp., especially VRE. *Enterococcus* spp. (*E. faecalis*, *E. faecium*, and unspeciated *Enterococcus* spp. grouped together) accounted for 5.4% of bacterial pathogens isolated by CANWARD from 2007 to 2016 (Table 1), making it the sixth most common pathogen in Canadian hospitals with VRE accounting for ∼6% of all *Enterococcus* spp. VRE are also commonly MDR with only linezolid and daptomycin as potential options for systemic infections. Linezolid, as mentioned earlier, is bacteriostatic; increasingly, isolates of VRE are intermediately resistant to linezolid and are associated with hematological toxicities with prolonged use. Daptomycin displays elevated MICs against some *Enterococcus* spp. and dosing (mg/kg) can be unclear.

### 2.2. Need for New Antimicrobial Agents for Infections Caused by Resistant Gram-Negative Bacilli

*E. coli*, *K. pneumoniae*, and *P. aeruginosa* are the most common Gram-negative bacilli causing infections in Canadian hospitals (Table 1) and are frequently MDR and are increasingly XDR. Growingly, ESBL-producing *E. coli* and *K. pneumoniae* are becoming resistant to carbapenems and isolates of carbapenem-resistant Enterobacteriaceae (CRE) are expected to increase in prevalence over time. The most immediate need for new antimicrobials to treat infections caused by Gram-negative pathogens in Canada is for infections such as urinary, respiratory, bacteremia (including endocarditis), intra-abdominal, and complicated skin and skin structure infections associated with ESBL-producing *E. coli* and *K. pneumoniae*, CRE, and MDR *P. aeruginosa*. The frequent and serious nature of neurotoxicity and nephrotoxicity with colistin, nephrotoxicity and ototoxicity with aminoglycosides, and increased mortality associated with tigecycline precludes their use unless absolutely necessary. *P. aeruginosa* is a nosocomial pathogen that is intrinsically resistant to many antimicrobial agents, frequently develops resistance on therapy, and is a concern because up to 10% of isolates are MDR, leaving only aminoglycosides (e.g., amikacin and tobramycin), colistin, and ceftolozane-tazobactam as therapies of last resort. Although ceftolozane/tazobactam is active against most MDR *P. aeruginosa*, new agents are needed, whether to be used alone or in combination with existing agents. Aminoglycosides, as mentioned earlier, are well-described nephrotoxins and ototoxins, require therapeutic drug monitoring, have poor activity at low pH, and demonstrate poor penetration into lung tissue, whereas colistin is a well-described neurotoxin and nephrotoxin. The risk/benefit ratio of using these either of these two agents is poor.

### 2.3. Ideal Properties of New Antimicrobial Agents

An ideal new antimicrobial agent for treating systemic infections in patients in Canadian hospitals would have a proven track record of bacteriological and clinical efficacy, an excellent safety profile, and outstanding pharmacokinetic (i.e., extensive tissue distribution including urine, bone, and cerebrospinal fluid (CSF)) and pharmacodynamics properties. The agent would be available as an intravenous therapy and possess a new mechanism of action with bactericidal activity against bacterial pathogens resistant to other antimicrobial classes (e.g., penicillins, cephalosporins, *β*-lactams/*β*-lactamase inhibitor combinations, carbapenems, fluoroquinolones, sulfonamides, and aminoglycosides). The agent would be associated with limited resistance development during treatment, would be available to be used alone or in combination with other antimicrobials, and would demonstrate additive or synergistic efficacy when used in combination with other agents.

## 3. Fosfomycin: Structure, Mechanism of Action, and Pharmacokinetic/Pharmacodynamic Properties

**Figure 1:**
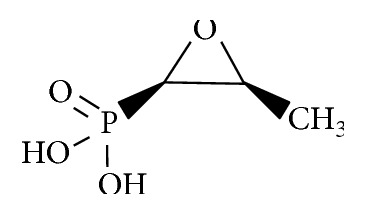
Chemical structures of fosfomycin.

The chemical structure of fosfomycin is *cis*-1,2-epoxypropyl phosphonic acid (C_3_H_7_O_4_P; molecular mass, 138.1 g/mol) and is depicted in Figure 1. The epoxide moiety is the principal structural determinant conferring fosfomycin's antibacterial activity. Fosfomycin was originally described in 1969, a result of screening broth cultures of soil containing *Streptomyces fradiae*. Today, fosfomycin is produced synthetically using phosphonic acid as starting material and is available commercially as a tromethamine salt (Monurol) for oral consumption and as a disodium salt for intravenous use. It is currently registered in 14 European countries, Japan, and various other courts and is undergoing clinical development in the US.

Fosfomycin has a unique chemical structure that is distinct from all other marketed classes of antimicrobial agents (i.e., *β*-lactams, glycopeptides, fluoroquinolones, macrolides, lincosamides, tetracyclines, and aminoglycosides). Fosfomycin has a low molecular mass, is freely soluble in water, has negligible plasma protein binding in vivo, and distributes predominantly into the extracellular space fluid of the body (approximately 0.30 L/kg body weight or a steady state volume of distribution is 18–27 L) [13–18]. Fosfomycin is able to penetrate into human lymphocytes where it elicits an effect against pathogens that have survived initial phagocytosis by neutrophils and increases intracellular killing of pathogens [19, 20].

Fosfomycin tromethamine has an oral bioavailability of 30–37% and a mean serum half-life of 5.7 h [13, 14]. After intravenous administration of a 4 to 8 g dose of fosfomycin, mean peak serum concentrations are commonly in the range of 200 to 400 *μ*g/mL [16, 21]. Fosfomycin is excreted predominantly in the urine in a nonmetabolized form [21]. Fosfomycin demonstrates greater antibacterial activity in weakly acidic environments (pH, 6.0). This property combined with its propensity for excretion as an active molecule in urine underlies its most common use as prophylaxis and treatment of urinary tract infections.

Fosfomycin exerts its antibacterial activity by inactivating UDP-*N*-acetylglucosamine-3-enolpyruvyl transferase (MurA), the enzyme responsible for ligating phosphoenolpyruvate (PEP) to the 3′-hydroxyl group of UDP-*N*-acetylglucosamine in the first step of peptidoglycan synthesis (conversion of UDP-*N*-acetylglucosamine to UDP-*N*-acetylmuramic acid) [13, 15, 16, 18, 22]. Fosfomycin is a PEP analog that inhibits MurA by alkylating an active site cysteine residue (Cys 115 in the *Escherichia coli* MurA enzyme). MurA is essential for any bacterium possessing muramic acid in its cell wall and accounts for fosfomycin's broad-spectrum activity and its bactericidal mechanism of action. In addition, fosfomycin has been demonstrated to reduce adherence of bacteria to uroepithelial cells. Fosfomycin accesses the bacterial cytosol primarily via the inducible hexose phosphate transport (UhpT) pathway but also gains entry using the constitutively expressed glycerol-3-phosphate transport (GlpT) pathway.

Limited data indicate that fosfomycin acts in a time-dependent manner with time above MIC (T > MIC) as the pharmacodynamic (PD) parameter associated with bacteriological efficacy [23]. Recent in vivo data (a neutropenic murine thigh infection model), however, published by Lepak et al. indicated that the AUC > MIC ratio was the pharmacodynamic parameter most closely linked to efficacy (*R*^2^, 0.70) in Gram-negative bacteria (*E. coli*, *K. pneumoniae*, and *P. aeruginosa*) [24]. AUC/MIC ratios associated with static effects against *E. coli*, *K. pneumoniae*, and *P. aeruginosa* were 24, 21, and 15, respectively [24]. Based on target attainment of 70–100%, comparable efficacy for all isolates tested by Lepak et al. was achieved using T > MIC [24]. At this time, it is not yet clear which fosfomycin PK/PD parameter is best associated with microbiological eradication, prevention of resistance, and clinical efficacy. As well, at this time, it is not yet known which pharmacodynamic targets should be achieved with fosfomycin and which doses should be used to achieve these PK/PD targets. There is also considerable evidence from animal models that fosfomycin can penetrate into biofilms and, in combination with other antimicrobial agents, possesses efficacy in eradicating a variety of biofilm-associated pathogens [25–28]. Recent data on fosfomycin's biofilm activity were reviewed by Falagas et al. [29].

## 4. Fosfomycin: Antimicrobial Susceptibility Testing

Standardized methods for antimicrobial susceptibility testing of fosfomycin are published by the Clinical and Laboratory Standards Institute (CLSI) [30] and the European Committee on Antimicrobial Susceptibility Testing (EUCAST) [31]. Most Canadian clinical microbiology laboratories follow CLSI guidelines. It is important to recognize that there are differences in the organisms for which fosfomycin MIC and zone diameter breakpoints apply in CLSI and EUCAST guidelines. As well, there are numerical differences in fosfomycin MIC and zone diameter breakpoints between CLSI and EUCAST methods, and these differences need to be considered when reading and comparing publications generated using different antimicrobial susceptibility testing methods.

Currently, CLSI-approved susceptibility breakpoints for fosfomycin exist only for *E. coli* and *E. faecalis*, with an MIC ≤64 *μ*g/mL (disk diffusion zone diameter: ≥16 mm) considered susceptible (resistance: MIC ≥256 *μ*g/mL; disk diffusion zone diameter: ≤12 mm); CLSI has only approved fosfomycin for testing isolates from urinary tract infections (acute uncomplicated cystitis following a single oral dose) [30]. EUCAST breakpoints for fosfomycin only exist for Enterobacteriaceae and staphylococci [31]. For Enterobacteriaceae, an MIC of ≤32 *μ*g/mL (for *E. coli* only: disk diffusion zone diameter ≥24 mm) is considered susceptible for both intravenous (systemic infections but may be dose dependent) and oral (uncomplicated urinary tract infection only) fosfomycin (resistance: MIC, >32 *μ*g/mL; for *E. coli* only: disk diffusion zone diameter, <24 mm) [31]. It is of note that agar dilution, not broth dilution, is the only approved MIC method for fosfomycin testing [30, 31] and that single colonies growing within the inhibitory zone of a disk diffusion test should be ignored as they do not indicate resistance [31]. Further EUCAST recommendations for disk diffusion zone diameter for *K. pneumoniae* are planned to be published in 2018. Further work is ongoing regarding *P. aeruginosa* and *S. aureus* (EUCAST, personal communication). For staphylococci, an MIC of ≤32 *μ*g/mL (disk diffusion zone size not available) is considered susceptible for intravenous (systemic infections) fosfomycin only (resistance, >32 *μ*g/mL); oral susceptibility breakpoints are not published by EUCAST for testing staphylococci against fosfomycin [31]. EUCAST breakpoints suggest that when considering the potential use of intravenous fosfomycin (i.e., the potential spectrum of activity of fosfomycin), isolates (genus/species) with MICs ≤32 *μ*g/mL may be considered susceptible and those with MICs >32 should likely be considered resistant. EUCAST also states that wild-type isolates of *P. aeruginosa* with MICs of ≤128 *µ*g/mL (epidemiological cutoff value) have been successfully treated with combinations of fosfomycin (oral and intravenous) and other agents. Therefore, in reviewing studies, the focus should be on MICs for fosfomycin and not necessarily on the reported percentages of isolates that are susceptible, and it is important to realize that depending on the IV dosage used of fosfomycin, greater target attainment can be achieved with greater fosfomycin daily doses. Also, studies reporting in vitro susceptibility testing of fosfomycin prior to 1983 should be disregarded as the importance of adding physiological concentrations of glucose-6-phosphate to testing media (to allow fosfomycin to exhibit its full antimicrobial activity) was unknown before that time [13]. Antimicrobial susceptibility testing (agar dilution or disk diffusion) requires agar supplementation with 25 *μ*g/mL of glucose-6-phosphate to ensure induction of the hexose phosphate transport (UhpT) pathway. Further, studies based upon broth dilution MIC testing should not be considered because of the relatively high likelihood of spontaneous mutation to fosfomycin resistance in broth [13].

Given the factors described in the preceding paragraphs, it is paramount to ensure that readers of susceptibility and clinical studies describing the activity of fosfomycin understand the parameters under which in vitro activity was defined. In this review, only MICs (and not susceptibility rates) are presented in the in vitro susceptibility testing data tables (Tables 2 and 3) describing the activity of fosfomycin against Gram-positive and Gram-negative bacteria.

## 5. Fosfomycin: Activity against Gram-Positive Bacteria

In general, fosfomycin possesses broad-spectrum activity against both Gram-positive and Gram-negative bacterial pathogens. The in vitro activity of fosfomycin against Gram-positive bacteria is summarized in Table 2 [13, 29, 31–34]. Fosfomycin is active against *S. aureus* (MIC_50_ 4 *μ*g/mL), including both MSSA and MRSA strains (Table 2). Fosfomycin is also active against *S. epidermidis* (MIC_50_ 4 *μ*g/mL) but displays limited activity against *S. saprophyticus* (MIC_50_ 64–128 *μ*g/mL). In the case of *Enterococcus* spp., fosfomycin displays activity against both *E. faecalis* and *E. faecium* (MIC_50_ 32–64 *μ*g/mL), including VRE (Table 2). In the case of *Streptococcus* spp., fosfomycin displays activity against *S. pneumonia*, *S. pyogenes*, and *S. agalactiae* with MIC_50_ 8–32 *μ*g/mL [31]. Some streptococci, corynebacteria, *Chlamydia*, and mycoplasmas are resistant to fosfomycin, likely due to the absence or low abundance of the MurA target.

## 6. Fosfomycin Properties: Activity against Gram-Negative Bacteria

The in vitro activity of fosfomycin against Gram-negative bacteria is summarized in Table 3 [13, 29, 31–35]; it includes activity against ESBL-producing and AmpC-producing Enterobacteriaceae (MIC_50_ 0.5–32 *μ*g/mL). The antibacterial spectrum of fosfomycin includes *Haemophilus* spp. (MIC_50_ 1 *μ*g/mL) and the majority of enteric Gram-negative bacteria but demonstrates higher MICs for *Morganella morgannii* (MIC_50_ 128–256 *μ*g/mL). Activity against *Klebsiella* and *Enterobacter* is variable (MIC_50_ 4–32 *μ*g/mL) (Table 3). Fosfomycin is moderately active against *P. aeruginosa* with variable MICs ranging from 4 to >512 *µ*g/mL (MIC_50_ 32–64 *μ*g/mL). *Acinetobacter* spp. (MIC_50_ 128 *μ*g/mL) and *Stenotrophomonas maltophilia* (MIC_50_ 64–128 *μ*g/mL) are poorly susceptible to fosfomycin. Gram-negative anaerobic bacteria are also not part of fosfomycin's antibacterial spectrum.

Activity against ESBL-producing pathogens, notably ESBL-producing *E. coli*, MDR *E. coli* [35], as well as ESBK and KPC-producing *K. pneumoniae* [36], is good to excellent (MIC_50_ 0.5–16 *μ*g/mL), because fosfomycin is not affected by cross resistance associated with mechanisms of resistance to other agents. Kaase et al. tested 80 isolates of Enterobacteriaceae with various carbapenemases (KPC, VIM, NDM, and OXA-48) and reported that 78% had MICs ≤32 *μ*g/mL and would thus be considered susceptible according to the EUCAST breakpoint [31, 37].

## 7. Fosfomycin: Resistance Mechanisms and Prevalence of Resistance

Resistance to fosfomycin most commonly arises via mutations in the chromosomal genes encoding the GlpT (primary mechanism of resistance) and UhpT (less common mechanism of resistance) pathways, thereby impeding fosfomycin's entry into bacterial cells and reducing access to its target site via the cytosol [22, 38–40]. Resistance to fosfomycin may also result less commonly from a myriad of other mechanisms including modification, inactivation, or overexpression of its target site enzyme (MurA), fosfomycin kinases (e.g., *fomA* and *fomB*), or inactivation via plasmid- and chromosomally encoded enzymes (e.g., *fosA*, *fosB*, and *fosX*) [16, 41, 42].

*Fos* enzymes are members of the glyoxalase superfamily and inactivate fosfomycin by catalyzing its conjugation with glutathione. These enzymes function by nucleophilic attack on carbon 1 of fosfomycin, which opens the epoxide ring and renders it ineffective. The enzymes differ by the identity of the nucleophile utilized in the reaction: glutathione for FosA, bacillithiol for FosB [43, 44], and water for FosX [40]. In general, FosA and FosX enzymes are produced by Gram-negative bacteria, whereas FosB is produced by Gram-positive bacteria [40]. Another enzyme, FosC, utilizes ATP and adds a phosphate group to fosfomycin, thus altering its properties and making the drug ineffective [45].

Resistance development during therapy is a confounding issue for fosfomycin [46, 47]. In vitro studies have shown that fosfomycin is associated with the development of resistance at a rate of one cell in every 10^6^ to 10^8^ cells [16, 18], thereby generating concerns regarding the emergence of resistance during therapy. However, these concerns have not been realized as the frequency of mutational resistance in vitro has not been observed in clinical studies or in settings of prolonged use, suggesting that there is a biological cost associated with such mutations (e.g., a decreased growth rate or, in the case of urinary tract infections, a reduced capacity for adherence) [16]. Data from the Antimicrobial Resistance Epidemiological Survey on Cystitis study showed that resistance to fosfomycin remained rare (∼2%) in regions where it was widely used [48]. Surveys of developing resistance patterns in Europe have also not revealed any major increases in plasmid-mediated resistance to fosfomycin [16]. Other possible explanations for fosfomycin's low resistance rate in urinary tract infections include its short contact time, high urine concentration (706 (±466) *µ*g/mL, 2–4 h after a single oral 3 g dose), and potentially higher compliance compared with agents dosed for 3–5 days. Lower frequencies of resistance development have been observed at higher fosfomycin concentrations and in media with an acidic pH [16]. In vitro resistance development in *E. coli* is less frequent than that in *K. pneumoniae* and *P. aeruginosa*, whereas relevant data for other Enterobacteriaceae are relatively scarce [15, 16].

Karageoropoulos et al. reported that resistance emerged in 2.3–6.7% of cases where fosfomycin was used for a clinical indication other than complicated urinary infections (e.g., in respiratory tract infections or osteomyelitis) apart from one study of suppurative otitis where the rate was 13.3% [16]. Considering clinical studies published before 2012, pathogens that most frequently arose as resistant to fosfomycin were *P. aeruginosa*, *Proteus* spp., *Klebsiella* spp., and *Enterobacter* spp. [16]. A more recent review undertook a similar investigation and reviewed 15 clinical studies that reported the use of intravenous fosfomycin in monotherapy and reported on resistance development during treatment [49]. In four of the 15 studies, no resistant isolate was identified after fosfomycin treatment. The remaining studies reported levels of resistance ranging from <3% to 17.9% [49]. In that review, the pooled estimate for resistance development during fosfomycin therapy was 3.4% (95% CI: 1.8–5.1%) [49]. Nonetheless, given that resistance to fosfomycin has the potential to develop in vivo when it is used as monotherapy, convention dictates that fosfomycin be administered as a component of combination therapy with one or more other antimicrobial agents when used for systemic therapy, except for the treatment of complicated urinary tract infections where it may be used as monotherapy [15, 50, 51].

## 8. Intravenous Fosfomycin: Clinical Trials and Clinical Utility

Few sufficiently powered, randomized, double-blind, placebo-controlled clinical trials comparing intravenous fosfomycin, alone or in combination, to a standard therapy can be found in the published literature. Instead, the published literature describing the use of intravenous fosfomycin, alone or in combination, is comprised of >1,000 case reports, case series, and cohort descriptive studies (summarized in [13, 15, 16, 29, 49, 52–54]); many of these studies are published in German, Spanish, French, Japanese, and Chinese. The limited number of robust clinical trials, in the English language scientific literature, aimed at determining the therapeutic and prophylactic value of using fosfomycin in combination with a second antimicrobial agent are listed in Tables 4 and 5 [55–58]. Additional, non-English language clinical trials have been summarized by Grabein et al. [49].

Grabein et al. recently performed a systemic review and meta-analysis of the clinical literature describing the use of intravenous fosfomycin from its inception to July 2016 [49]. Their meta-analysis summarized the utility of intravenous fosfomycin in >100 published studies involving >5,000 patients [49]. The majority of studies (∼90%, 113/128 studies) available for review were conducted in European countries or Japan and 84 of the 128 studies were published before 1989 [49]. Approximately 50% of the studies were retrospective case series, with only a minority of studies (6%; 8/128 studies) being randomized controlled trials [49]. The randomized controlled trials comparing intravenous fosfomycin therapy (either monotherapy or combination therapy) against another therapy regimen were generally of only poor or fair quality due to a lack of statistical power or the presence of possible confounding variables [49]. No difference in clinical (odds ratio 1.44) or microbiological efficacy (odds ratio 1.28) between intravenous fosfomycin and other antimicrobial agents was observed in comparative trials [49]. Grabein et al. also reported that, in general, intravenous fosfomycin demonstrated a favorable safety profile, with only mild adverse effects (e.g., mild hypokalemia due to high sodium load with administration of fosfomycin disodium) not requiring discontinuation of therapy [49]. Other reviews and studies have reported similar conclusions [13, 15, 16, 51, 52, 59].

More recently, in 2017, Zavante Therapeutics completed a Phase III clinical trial through the U.S. FDA (ClinicalTrials.gov identifier: NCT02753946) that studied the safety and efficacy of intravenous fosfomycin 6 g every 8 h (ZTI-01) versus piperacillin-tazobactam 4.5 g every 8 h (each administered for 7 days) for the treatment of complicated urinary tract infections or acute pyelonephritis in hospitalized patients (details of this study discussed later). Two other Phase III clinical trials are currently registered in ClinicalTrials.gov (U.S. National Library of Medicine) that involve intravenous fosfomycin and are currently recruiting participants. The first is a study of fosfomycin versus meropenem for the treatment of bacteremic patients with urinary tract infection due to multidrug-resistant *E. coli* (ClinicalTrials.gov identifier: NCT02142751), and the second is a study to determine whether the combination of daptomycin and fosfomycin is superior to daptomycin alone in the treatment of MRSA bacteremia (ClinicalTrials.gov identifier: NCT01898338).

Intravenous fosfomycin will likely be used in combination with an antimicrobial agent belonging to another class (e.g., *β*-lactams, aminoglycosides, fluoroquinolones, glycopeptides, or glycolipopeptides) when used to treat serious or systemic infections outside of the urinary tract. The combination partner of intravenous fosfomycin would be selected according to indication and patient's individual clinical situation. Fosfomycin can be combined with all other antimicrobial classes according to type of infection and causative pathogens. The rationale for combining fosfomycin with a second agent is primarily based on preventing the emergence of fosfomycin resistance [60] and broadening the antimicrobial spectrum. Combination therapy may also offer additive or synergistic activity/efficacy and attractive pharmacokinetic properties for difficult to reach compartments [61]. The true benefit of adding fosfomycin to another antimicrobial agent for patient therapy is poorly described as published clinical studies are rarely structured to compare fosfomycin and the other agent alone and then in combination. Of interest, combination therapy data with fosfomycin in biofilm animal models have shown better results compared to fosfomycin monotherapy [25, 26]. Experts suggest that the use of the highest dose of fosfomycin should be considered in the treatment of systemic infections to combat the potential for resistance development [16].

As mentioned earlier, fosfomycin allocates extensively into extracellular space fluid [13] and distributes rapidly into tissues [15, 62], achieving clinically relevant concentrations in sites such as serum, soft tissues, lung, bone, CSF, abscess fluid, and endocardial tissue. Fosfomycin's rapid penetration into tissues is a highly desirable characteristic for the treatment of serious infections. The highest peak concentrations of fosfomycin are achieved in serum and urine following intravenous administration [15, 52], whereas the concentrations of fosfomycin achieved in lung (50–60% of serum concentration), bone (50%), CSF (20–50%), soft tissues (75%), and other tissues are lower than those in serum [62–64].

The case reports, case series, cohort descriptive studies, and clinical trials describing the use of fosfomycin in combination with other antimicrobial agents can be grouped into studies of CNS infections, respiratory infections, complicated urinary tract infections, infectious endocarditis and septicemia, osteomyelitis, and soft tissue infections. Overall, cure rates of >80% have been observed [15, 29, 49, 52].

### 8.1. CNS Infections

Fosfomycin can pass through the blood-brain barrier in both healthy and inflamed meninges [23]. Inflammation increases the penetration rate of fosfomycin across the meninges [23]. Fosfomycin crosses the blood-brain barrier independent of inflammation and reaches therapeutic concentrations within the CSF within 2 h of receiving an 8-g intravenous dose [23]. In the area of CNS infections (meningitis), a number of studies have shown the efficacy of fosfomycin combined with an aminoglycoside or cefotaxime in the treatment of meningitis due to Gram-negative bacilli (*H. influenzae* and *E. coli*), *Neisseria meningitidis*, *S. aureus*, and, recently, pneumococci with reduced susceptibility to penicillin (MIC, >0.1 *µ*g/mL) [17]. In patients who received a 5-g dose, three times daily, fosfomycin concentrations of >30 *μ*g/mL were reached in the CSF by the second day of treatment [17]. Fosfomycin may have utility in the treatment of ventricular shunt infections where the pathogens are frequently resident flora of skin and mucus membranes (e.g., coagulase-negative staphylococci). These infections are known to cause only minor inflammatory changes at the blood-brain barrier, making fosfomycin an attractive therapy, given its ability to cross intact meninges and achieve therapeutic concentrations.

### 8.2. Respiratory Tract Infections

In the treatment of respiratory tract infections (nosocomial pneumonia), particularly when *P. aeruginosa* and *S. aureus* are pathogens, combinations of fosfomycin with a ureidopenicillin or ceftazidime (for *P. aeruginosa*) or vancomycin (for *S. aureus*) have proven effective. A 1986 randomized controlled trial compared the effectiveness of intravenous fosfomycin (4 g every 8 h) versus gentamicin (80 mg every 8 h), both combined with ampicillin in critically ill patients with pneumonia [56]. Relatively high clinical success rates were observed in both treatment groups (94% (*n*=17) versus 80% (*n*=15)). When dosing fosfomycin at 2 g, serum concentrations attained were 32 *μ*g/mL, 1–2 h post-intravenous administration; lung concentrations were 32–52% of corresponding serum concentrations [65]. When dosed at 4 g, fosfomycin serum concentrations were 243 *μ*g/mL, 1 h following intravenous administration; lung concentrations were 54% and 44% of corresponding serum concentrations in healthy and infected lung tissue, respectively [66].

The place of fosfomycin in the treatment of *P. aeruginosa* infections is still poorly established [29, 53, 67]. However, favorable results for combinations of fosfomycin with an aminoglycoside have been reported in cases of cystic fibrosis in children with episodes of superinfection involving *P. aeruginosa*.

### 8.3. Complicated Urinary Tract Infections

Severe urinary tract infections have been treated with fosfomycin monotherapy in view of the excellent urinary elimination of this agent [16]. Favorable results have been obtained in severe pyelonephritis (various doses/routes used), with sterilization of the urine obtained in 79% of cases at the end of treatment (7 days) [16]. The lack of nephrotoxicity of fosfomycin in patients suffering from parenchymal renal infection, together with the antibacterial spectrum of fosfomycin (i.e., includes enteric Gram-negative bacilli), suggests reliable efficacy in the treatment of these infections [68]. Several reviews of studies have suggested that fosfomycin is in association with favorable outcomes (versus comparators) when treating with MDR Gram-negative bacterial infections, predominantly urinary tract infections [16, 51–53, 60].

More recently, in 2017, the ZEUS study, a multicentered, randomized, double-blind phase II/III study of hospitalized patients with complicated urinary tract infections or pyelonephritis received a 1-h infusion of 6 g fosfomycin every 8 h (18 g total daily dose) or a 1-h infusion of 4.5 g piperacillin-tazobactam every 8 h (13.5 g total daily dose) for 7 days [51]. Patients with concurrent bacteremia received 14 days of treatment. Oral step-down therapy was prohibited. Overall success rates for patients with complicated urinary tract infections or acute pyelonephritis were numerically higher for fosfomycin (64% or 119/164) than for piperacillin-tazobactam (54.5% or 97/178). Overall success rates for patients with complicated urinary tract infections were numerically higher for fosfomycin (64%) than for piperacillin-tazobactam (42%), whereas for acute pyelonephritis, success rates were similar between treatment groups, 68% (fosfomycin) versus 66% (piperacillin-tazobactam) [51]. Microbiological eradication rates were numerically higher in the fosfomycin group than in the piperacillin-tazobactam group (66% versus 56%), whereas clinical cure rates were similar between treatment groups, 91% (fosfomycin) versus 92% (piperacillin-tazobactam) [51]. IV fosfomycin was well tolerated with most frequent treatment-related adverse effects being gastrointestinal in nature along with hypokalemia (primarily mild) due to the high sodium load of administering fosfomycin disodium.

### 8.4. Infectious Endocarditis

Fosfomycin is recommended for the treatment of staphylococcal endocarditis as an alternative therapy by the current European Society of Cardiology guideline for the management of infective endocarditis [69]. Del Rio et al. evaluated the efficacy of fosfomycin in combination with imipenem in a multicentered trial including 12 patients with infective endocarditis, who had previously received unsuccessful treatment with other antimicrobial regimes [70]. The authors reported a clinical success rate of 69% and only one death related to MRSA infection [70]. Furthermore, negative blood cultures were achieved in all cases 72 h after treatment [70]. The combination of fosfomycin and an aminoglycoside has been found to be consistently rapidly bactericidal (95%) [71] and effective in treating staphylococcal septicemia [72, 73]. In endocarditis, the results of use of fosfomycin vary: poor in the case of viridans group streptococci (which may not be part of the antibacterial spectrum of fosfomycin) but more favorable in staphylococcal endocarditis, where the combination of fosfomycin with an aminoglycoside for *S. aureus* or with a fluoroquinolone for MRSA has demonstrated efficacy [73]. In *Staphylococcus epidermidis* infections, the limited number of active antibacterial agents may promote fosfomycin to a position of choice, given it is combined with vancomycin, an aminoglycoside, or a fluoroquinolone. In *P. aeruginosa* septicemia, cure rates on the order of 83% have been reported [74].

### 8.5. Osteomyelitis

There is considerable clinical experience using fosfomycin in combination regimens for various types of bone infections, primarily complicated fractures and osteomyelitis or septic arthritis in children [17]. Fosfomycin penetrates well into osseous tissue. After a 5 g or 10 g intravenous dose, fosfomycin concentrations in bone and interstitial space fluids were 117–119 *μ*g/mL and 368–451 *μ*g/mL, respectively [62, 75]. Because of its good diffusion into bone, fosfomycin has the potential to be recommended in the treatment of staphylococcal bone and joint infections (osteomyelitis). For this indication, fosfomycin is recommended to be combined with oxacillin (for MSSA) in chronic osteomyelitis or with an aminoglycoside or fluoroquinolone in septicemia with an osteoarticular localization [62, 75].

### 8.6. Skin and Soft Tissue Infections

There are also clinical data to support the use of fosfomycin in the treatment of soft tissue infections. In diabetic foot infections, median fosfomycin tissue concentrations of ≥22–25 *μ*g/mL were measured (200 mg/kg of body weight corresponding to 3 doses × 5 g fosfomycin/day); measured concentrations were comparable for inflamed and noninflamed tissues [63]. A multicenter study has evaluated treatment with fosfomycin (8–24 g daily) in combination with a conventional agent for patients with limb-threatening diabetic foot infections. Limb preservation was achieved in the great majority (48/52) of patients [76]. Legat et al. also reported that a daily dose of 15 g of fosfomycin (3 doses × 5 g) maintained sufficient fosfomycin concentrations in the interstitial space fluid of inflamed tissues to inhibit the growth of relevant bacteria, such as *S. aureus*, when treating cellulitis and diabetic foot infections [63].

## 9. Summary of Fosfomycin Properties

Fosfomycin inhibits cell wall synthesis, resulting in bactericidal activity using a mechanism different from that of *β*-lactams. It is a broad-spectrum agent with activity against a variety of Gram-positive and Gram-negative bacteria. Fosfomycin's unique mechanism of action allows it to be active against organisms resistant to a variety of other antimicrobial classes, including MRSA, VRE, MDR *E. coli*, MDR *Klebsiella* spp., and some MDR *P. aeruginosa*. Studies assessing potential for resistance demonstrate that fosfomycin resistance is associated with a biological cost to the pathogen making resistant subpopulations less fit for *E. coli* but not for *P. aeruginosa* [77]. Fosfomycin when used in combination with a variety of antimicrobials (e.g., *β*-lactams, carbapenems, and aminoglycosides) has demonstrated in vitro additivity and even synergistic activity. Fosfomycin due, in part, to its low molecular weight achieves extensive tissue distribution including difficult to reach compartments such as aqueous humor, vitreous humor, abscess fluid, and CSF.

Clinically, intravenous fosfomycin has been used to treat a variety of infections including: urinary tract infections, gastrointestinal infections, meningitis, pulmonary infections including cystic fibrosis, endocarditis, ocular infections, postoperative wound infections, osteomyelitis, obstetric and gynecological infections, intra-abdominal infections, diabetic foot infections, shunt infections, and bloodstream infections [13, 15, 16, 29, 49, 52–54]. The most common bacteriologic infectious causes treated by fosfomycin have been MDR *S. aureus*, MDR *S. epidermidis*, antimicrobial-resistant Enterobacteriaceae, and MDR *P. aeruginosa*. Frequently, fosfomycin was administered to patients after initially failing first- and sometimes second-line antimicrobials and was frequently used in combination with other antimicrobial agents such as *β*-lactams, fluoroquinolones, aminoglycosides, vancomycin, and rifampin. Clinical resolution of infections with fosfomycin treatment occurred in ∼80% of treated patients.

Intravenous fosfomycin has been available and used extensively in a variety of countries for over 40 years and has demonstrated exceptional safety including in neonates and children, even with long-term administration (weeks). Adverse effects, which are not common and mild in nature, are primarily gastrointestinal in nature and as well hypokalemia. Available data suggest that to date, no clinically relevant pharmacological interactions between fosfomycin and other agents, including drugs, stimulants, or food, have been reported.

## 10. Role of Intravenous Fosfomycin in Canada

The availability of intravenously administered fosfomycin in Canada revolves primarily around its potential use in treating hospitalized patients with MDR Gram-positive and MDR Gram-negative infections not responding to other antimicrobial agents and/or for patients who cannot tolerate first- and/or second-line agents due to adverse effects. With MRSA rates in Canada at ∼20%, VRE rates at 6% and rising, rates of ESBL-positive *E. coli* and *Klebsiella* spp. at ∼12% and ∼7%, respectively, and rising, and MDR *P. aeruginosa* at 6.5%, fosfomycin is needed for use in combination with standard antimicrobials in place of toxic drugs such as aminoglycosides, tigecycline, and colistin.

Intravenous fosfomycin was recently added by the World Health Organization (WHO) to its list of essential medicines for treatment of adults and children. It is listed among the WHO's RESERVE group of antibacterial medicines as a treatment option for highly specific patients and settings when other alternatives would be inadequate (e.g., biofilm-related infections, deep-seated focus, abscess formation, severe allergy, resistance to standard therapy, severe clinical condition, presence of comorbidities, or intolerability of standard therapy) or have already failed (e.g., serious life-threatening infections due to MDR bacteria). Other members of the RESERVE group are aztreonam, cefepime, ceftaroline, polymyxin B, colistin, linezolid, tigecycline, and daptomycin.

Intravenous fosfomycin's role in Canadian hospitals would be as therapy for patients with infections who have not responded to first- and potentially second-line antimicrobials or in patients who cannot tolerate (due to adverse effects) first- and second-line antimicrobials. Intravenous fosfomycin would primarily be used in combination with *β*-lactams, carbapenems, fluoroquinolones, aminoglycosides, and glycopeptides/glycolipopeptides for the treatment of MDR MRSA, MDR methicillin-resistant *S. epidermidis* (MRSE), MDR VRE, MDR Enterobacteriaceae, and MDR *P. aeruginosa* infections. Using fosfomycin in combination with another antimicrobial should limit the development of resistance to this agent over time. Because of its proven safety, it should be considered for use in preference to last line toxic agents such as colistin, tigecycline, and aminoglycosides. In addition, due to its exceptional tissue distribution it could be used not only for the most common infections such as bacteremia, urinary tract, skin and soft tissue, and respiratory infections but also for difficult to treat infections such as bone infections, meningitis, and invasive ocular infections. Finally, based upon the results of the ZEUS study, IV fosfomycin would be a preferred option for treating cUTI due to resistant Enterobacteriaceae.

## Figures and Tables

**Table 1:** The 20 most common bacterial pathogens isolated from patient samples in Canadian hospitals, CANWARD surveillance study, 2007–2016.

Rank	Organism	Number of isolates	% of total isolates
1	*Escherichia coli*	8,387	19.5
2	*Staphylococcus aureus*, MSSA	7,146	16.6
3	*Pseudomonas aeruginosa*	3,864	9.0
4	*Streptococcus pneumoniae*	2,626	6.1
5	*Klebsiella pneumoniae*	2,624	6.1
6	*Staphylococcus aureus*, MRSA	1,963	4.6
7	*Haemophilus influenzae*	1,764	4.1
8	Coagulase-negative staphylococci/*Staphylococcus epidermidis*	1,616	3.8
9	*Enterococcus faecalis*	1,255	2.9
10	*Enterobacter cloacae*	1,060	2.5
11	*Streptococcus agalactiae*	689	1.6
12	*Stenotrophomonas maltophilia*	669	1.6
13	*Klebsiella oxytoca*	668	1.6
14	*Proteus mirabilis*	647	1.5
15	*Serratia marcescens*	647	1.5
16	*Streptococcus pyogenes*	642	1.5
17	*Enterococcus*, nonspeciated	582	1.4
18	*Enterococcus faecium*	481	1.1
19	*Candida albicans*	478	1.1
20	*Moraxella catarrhalis*	468	1.1
	Others	4,729	11.0
Total		42,938	

Adapted from [6].

**Table 2:** In vitro activity of fosfomycin against aerobic and facultative Gram-positive bacteria (cumulative data from [13, 29, 31–34]).

Organism	Fosfomycin			
	Number of isolates tested	MIC_50_ (*µ*g/mL)	MIC_90_ (*µ*g/mL)	Range (*µ*g/mL)
*Enterococcus faecalis*	1965	32–64	64	0.5–512
*Enterococcus faecium*	620	32–64	64–128	0.5–128
*Enterococcus* spp.	137	16–32	64	0.25–>256
*Staphylococcus aureus*	2213	4	16	0.12–512
*Staphylococcus aureus*, MSSA	103	4	4	0.5–16
*Staphylococcus aureus*, MRSA	263	4	8–64	0.5–512
*Staphylococcus epidermidis*	896	8	128	0.5–256
*Staphylococcus saprophyticus*	309	64–128	256–>512	2–>512
*Streptococcus pneumoniae*	57	8	16	4–32
*Streptococcus pyogenes*	150	32	64	2–64
*Streptococcus agalactiae*	154	8–32	64	1–64

**Table 3:** In vitro activity of fosfomycin against aerobic and facultative Gram-negative bacteria (cumulative data from [13, 29, 31–35]).

Organism	Number of isolates tested	Fosfomycin		
		MIC_50_ (*µ*g/mL)	MIC_90_ (*µ*g/mL)	Range (*µ*g/mL)
*Acinetobacter* spp.	244	128	128–512	0.25–512
*Citrobacter* spp. (*C. diversus*, *C.freundii*, and *C. koseri*)	437	0.5–2	1–4	≤0.12–64
*Enterobacter* spp. (*E. agglomerans*, *E. aerogenes*, and *E. cloacae*)	808	8–32	16–256	0.25–>512
*Escherichia coli*	7735	0.5–4	1–16	0.25–512
*Escherichia coli* ESBL-producing	296	2	4	≤1–512
*Escherichia coli* AmpC-producing	135	2	4–16	≤1–>512
*Haemophilus influenzae*	50	1	4	1–128
*Klebsiella oxytoca*	153	8	16–32	1–64
*Klebsiella pneumoniae*	284	4–16	16–128	≤2–512
*Klebsiella* spp.	788	16	32–128	≤2–512
*Morganella morganii*	59	128–256	512	8–>512
*Proteus mirabilis*	1533	1–4	8–64	≤0.12–>512
*Proteus vulgaris* (indole-positive *Proteus*)	431	≤2–16	8–256	0.5–256
*Providencia* spp. (*P. rettgeri* and *P. stuartii*)	164	2–16	8–128	≤2–512
*Pseudomonas aeruginosa*	1450	32–64	64–128	2–>512
*Pseudomonas* spp.	35	128	256	≤0.5–512
*Serratia marcescens*	383	8	16–32	0.5–128
*Shigella* spp.	185	2	2	0.5–64
*Stenotrophomonas maltophilia*	151	64–128	128	16–512

**Table 4:** Comparative clinical studies describing outcomes for patients receiving intravenous fosfomycin in combination with a second antimicrobial agent.

Trial design (reference) Infection type (*n*)	Treatment regimens and outcomes	Pathogens
Open label nonrandomized [55] Septicemia (32), osteoarthritis (1), meningitis (1), and pulmonary infection (1)	Group 1: 17 patients fosfomycin (237.1 mg/kg daily IV)/penicillin Cure: 16/17 (94.1%) Group 2: 18 patients gentamicin/penicillin Cure: 14/18 (77.7%)	MSSA

Prospective randomized [56] Ventilator-associated pneumonia (22) and pneumonia (10)	Group 1: 17 patients fosfomycin (12 g daily IV)/ampicillin Cure: 10/17 (58.8%); improvement: 6/17 (35.2%) Group 2: 15 patients gentamicin/ampicillin Cure: 7/15 (46.6%); improvement: 5/15 (33.3%)	*E. coli*, coagulase-positive staphylococci, *P. aeruginosa*, and *Klebsiella*

**Table 5:** Comparative clinical studies describing outcomes for patients receiving prophylaxis with intravenous fosfomycin in combination with a second antimicrobial agent.

Trial design (reference) Operation type	Treatment regimens and outcomes
Multicenter, double-blind, randomized [57] Elective colorectal surgery	Group 1: 259 patients received fosfomycin (8 g IV)-metronidazole before operation and second infusion of fosfomycin (8 g IV) 8 h later Abdominal infection in 12/259 (4.6%) of patients; pneumonia in 13/259 patients (5.0%) Group 2: 258 patients received doxycyline-metronidazole before operation and second identical infusion 8 h later Abdominal infection in 19/258 (7.4%) of patients; pneumonia in 5/258 patients (2.0%)

Prospective double-blind randomized [58] Elective colorectal surgery	Group 1: 72 patients, 3 days before operation received placebo, 1 h before operation received fosfomycin-metronidazole (8 g IV) Infective complications in 9/72 (12.5%) of patients Group 2: 77 patients, 2 days before operation received bacitracin-neomycin, 1 day before operation received metronidazole, 1 h before operation received ampicillin Infective complications in 8/77 (10.4%) of patients
